# Super-Coulombic atom–atom interactions in hyperbolic media

**DOI:** 10.1038/ncomms14144

**Published:** 2017-01-25

**Authors:** Cristian L. Cortes, Zubin Jacob

**Affiliations:** 1 Department of Electrical and Computer Engineering, University of Alberta, Edmonton, Alberta, Canada T6G 2V4; 2 Birck Nanotechnology Center and Purdue Quantum Center, School of Electrical and Computer Engineering, Purdue University, 1205 West State Street, West Lafayette, Indiana 47906, USA

## Abstract

Dipole–dipole interactions, which govern phenomena such as cooperative Lamb shifts, superradiant decay rates, Van der Waals forces and resonance energy transfer rates, are conventionally limited to the Coulombic near-field. Here we reveal a class of real-photon and virtual-photon long-range quantum electrodynamic interactions that have a singularity in media with hyperbolic dispersion. The singularity in the dipole–dipole coupling, referred to as a super-Coulombic interaction, is a result of an effective interaction distance that goes to zero in the ideal limit irrespective of the physical distance. We investigate the entire landscape of atom–atom interactions in hyperbolic media confirming the giant long-range enhancement. We also propose multiple experimental platforms to verify our predicted effect with phonon–polaritonic hexagonal boron nitride, plasmonic super-lattices and hyperbolic meta-surfaces as well. Our work paves the way for the control of cold atoms above hyperbolic meta-surfaces and the study of many-body physics with hyperbolic media.

## Abstract (web summary)

Dipole-dipole interactions give rise to a number of physical phenomena, but they are typically limited to the Coulombic near-field. Here authors demonstrate the existence of a class of real- and virtual-photon interactions which have a singularity in media with hyperbolic dispersion.

Dipole–dipole interactions (DDIs) are instrumental in mediating entanglement and superradiance in cold atoms^1^,^2^,^3^, as well as coherent coupling between single molecules or atoms^4^,^5^,^6^,^7^. Often described by real and virtual photon exchange, they also cause cooperative frequency shifts between superconducting qubits in circuit quantum electrodynamic (QED) systems^8^,^9^ and Förster resonance energy transfer (FRET) between dye molecules or quantum dots^10^,^11^. There are two fundamental ways of controlling the strength and length scales of DDIs. The first method involves the tuning of intrinsic atomic properties such as transition dipole moments and transition frequencies (*cf*. highly excited Rydberg atoms and superconducting qubits^7^,^12^,^13^). The second method involves the tuning of the QED vacuum, achieved through cavities, waveguides and photonic bandgaps^14^,^15^,^16^,^17^. Up to now, these electrodynamic methods have relied on resonant effects that require large quality factors along with extensive nanofabrication steps. It is an open question, however, whether there exists alternative non-resonant techniques for controlling DDIs that would be robust to broad spectral lineshapes of atoms or molecules with possible room temperature applications. Here we present work related to this new avenue of research.

In this study, we reveal a class of divergent excited-state atom–atom interactions that can occur in natural and artificial media with hyperbolic dispersion. Unlike the above mentioned approaches, which engineer radiative coupling, we show that the homogeneous hyperbolic medium itself fundamentally alters the Coulombic near-field. The resultant singular long-range interaction, referred to as a super-Coulombic interaction, is described by an effective interaction distance that goes to zero (*r*_e_→0) along a material-dependent resonance angle. We show that this interaction affects the entire landscape of real photon and virtual photon phenomena such as the cooperative Lamb shift (CLS), the cooperative decay rate (CDR), resonance energy transfer rates and frequency shifts, as well as resonant interatomic forces. Although we find that the singularity is curtailed by material absorption, it still allows for interactions with much larger magnitudes and longer ranges than those found in any conventional media. We also show that atoms in a hyperbolic medium will exhibit a strong orientational dependence that can effectively switch the dipolar interaction off or on, providing an additional degree of freedom to control DDI. Our investigation reveals a marked contrast between ground-state and excited-state interactions which can be used to distinguish the super-Coulombic effect in experiment. Finally, we provide a unified perspective for controlling DDIs on multiple experimental platforms for hyperbolic media including plasmonic super-lattices, hyperbolic metasurfaces and natural hyperbolic media such as hexagonal boron nitride (h-BN).

We emphasize that the materials platform we introduce in this study, to enhance DDIs, is fundamentally different from the cavity QED^18^,^19^ or waveguide QED regimes^5^,^8^,^20^,^21^ (see Supplementary Table 1). We do not rely on atom confinement^2^,^5^,^6^,^7^,^19^, cavity resonances or modal effects such as the quasi transverse electromagnetic (TEM) mode in circuit QED^8^, the band-edge slow light as in PhC waveguides^5^,^6^,^22^, the low-mode volume of plasmonic waveguides^21^,^23^ or the infinite phase velocity at the cutoff frequency of epsilon-near-zero (ENZ) waveguides^14^,^24^. We also stress that the super-Coulombic effect engineers the conventional non-radiative (longitudinal) near-fields as opposed to radiative (transverse) modes and will occur over a broad range of frequencies due to the broadband nature of the hyperbolic dispersion relation^25^,^26^,^27^,^28^. Figure 1 depicts a schematic of the proposed super-Coulombic DDI using h-BN^29^,^30^,^31^,^32^ and two dopant atoms. In the infrared spectral range, h-BN is a uniaxial material that supports ordinary waves (polarization perpendicular to the optic axis) and extraordinary waves (polarization along the optic axis). Extraordinary waves satisfy the hyperbolic dispersion relation 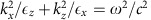 when 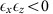.

## Results

### QED theory of hyperbolic media

We begin by formulating the QED theory^33^ of DDIs between two neutral, non-magnetic atoms in a hyperbolic medium. We focus on dipolar interactions where the electrodynamic field is initially prepared in the vacuum state 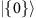. Using the multipolar Hamiltonian, the interaction of two neutral atoms [positions **r**_*j*_, transition frequencies *ω*_*j*_ and transition electric dipole moments 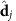 (*j*=*a*, *b*)] is specified by the interaction Hamiltonian


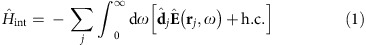


where h.c. stands for the Hermitian conjugate. The matter-assisted electric field is given by


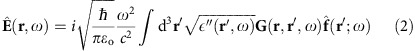


and **G**(**r**, **r**′;*ω*) is the classical dyadic Green function that satisfies the macroscopic Maxwell equations. Here, 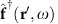 and 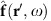 are bosonic field operators, which play the role of the creation and annihilation operators of the matter-assisted electromagnetic (polaritonic) field. The unique interaction properties are a direct result of the dispersion relation of the hyperbolic polariton, as opposed to the photonic dispersion relation, *ω*=*ck*, seen in vacuum. The electric field is defined so that it rigorously satisfies the equal-time commutation relations and fluctuation–dissipation theorem^33^. We use conventional perturbation theory to calculate the various dipolar interactions in a hyperbolic medium. We emphasize that the QED theory captures both ground state–ground state interactions and excited state–ground state interactions, which a semiclassical approach cannot.

### Resonant dipole–dipole interaction

If the initial state of the atomic system is prepared in the symmetric or anti-symmetric state, 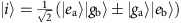, then one can show that the resonant DDI (RDDI) (see Methods) is given by


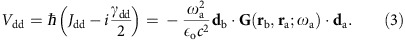


where 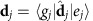 is the transition dipole moment of atom *j*, assumed to be real. *J*_dd_ is the CLS (also known as the virtual photon exchange interaction) and *γ*_dd_ is the CDR commonly associated with superradiant or subradiant effects.

Our result for the RDDI in a hyperbolic medium 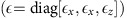 is


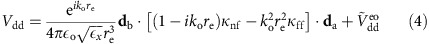


valid when **r**_a_≠**r**_b_. The first term arises exclusively from extraordinary waves following a hyperbolic dispersion, whereas the second term 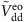 arises from a combination of ordinary and extraordinary waves. Here we have defined the near-field and far-field dipole orientation matrix factors 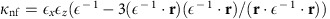 and 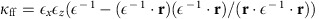, respectively. Equation (4) reduces to the vacuum RDDI expression when 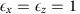, which is applicable both in the retarded (*r*≫*λ*) and non-retarded (*r*<<*λ*) regimes. The most unique aspect of DDIs in uniaxial media is the divergence that is predicted from the first term only when the hyperbolic condition 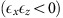 is satisfied. In the ideal lossless limit, we find that the effective interaction distance between two atoms, 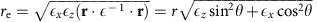, tends towards the limit


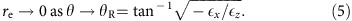


This super-Coulombic effect results in the divergence of the DDI strength |*V*_dd_|/*ħ* along the resonance angle *θ*_R_, defined with respect to the optic axis.

Atoms in a hyperbolic medium will then have an associated CLS and CDR


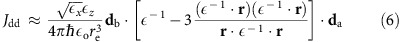



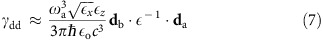


in the limit *θ*→*θ*_R_. Equations (6) and (7) are the dominant factors of the extraordinary wave contribution only.

We now contrast the scaling of CLS with distance when mediated by hyperbolic media as opposed to vacuum modes. In vacuum, for separation distances much larger than the transition wavelength, the CLS scales as 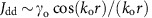 and becomes much smaller than the free-space spontaneous emission rate (*γ*_o_). On the other hand, for distances much smaller than the wavelength, the CLS scales as 
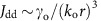
, which implies that it can become much larger than the spontaneous emission rate. In contrast, the CLS in a hyperbolic medium is dependent on 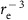, 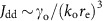, for all interatomic distances. The material-dependent factor 1/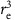 diverges in the lossless case and therefore results in giant CLSs for short and large interatomic distances.

This marked contrast is also revealed in the CDR. At large distances, the CDR in vacuum scales as 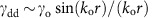, therefore becoming weak for distances much larger than the wavelength. For distances much smaller than the wavelength, the CDR becomes independent of position, 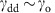, and remains on the order of the free-space spontaneous emission rate. In contrast, the CDR in a hyperbolic medium along the resonance angle is not dependent on the effective interaction distance *r*_e_ and instead it depends crucially on the orientation angle *φ* of the dipoles, 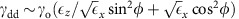. When both dipoles are oriented perpendicular to the optic axis (*φ*=*π*/2), there exists a unique wavelength when the medium can achieve an anisotropic ENZ medium (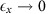 and 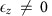) resulting in a divergent CDR. Surprisingly, the effect is independent of interatomic distance. When both dipoles are parallel to the optic axis (*φ*=0), the same anisotropic ENZ condition gives a null CDR between the two atoms, independent of interatomic distance.

We will now consider the role of material absorption (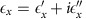 and 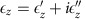) on atom–atom interactions in a hyperbolic medium. We find that the effective interaction distance is not zero and tends to the finite value 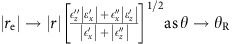. This curtails the singularity of the hyperbolic dipolar interaction but nevertheless allows for very large interaction strengths compared with conventional media whenever |*r*_e_|/|*r*|<1 is satisfied. Material absorption will also modify the spatial scaling laws of the RDDI in equation (3) so that both the CDR and Lamb shift will scale as 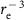. Another consequence of material absorption on RDDIs is in the transition from non-retarded (*r*^−3^) to retarded (*r*^−1^) interactions. In vacuum, the transition occurs when the interatomic separation distance is on the order of wavelength,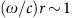. In an ideal lossless hyperbolic medium, this transition from near-field to far-field does not occur, as the effective separation distance approaches zero, *r*_e_→0 specifically along the resonance angle of a hyperbolic medium. Therefore, we find that RDDI should scale with the characteristic power law of near-field (longitudinal) non-radiative interactions (*r*^−3^) for all interatomic distances. Once material absorption is included, the transition is expected to occur approximately when 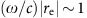. The dipolar interactions will transition from the power law 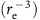 to the exponential scaling law 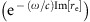, which is valid at large interatomic distances.

**Figure 2:**
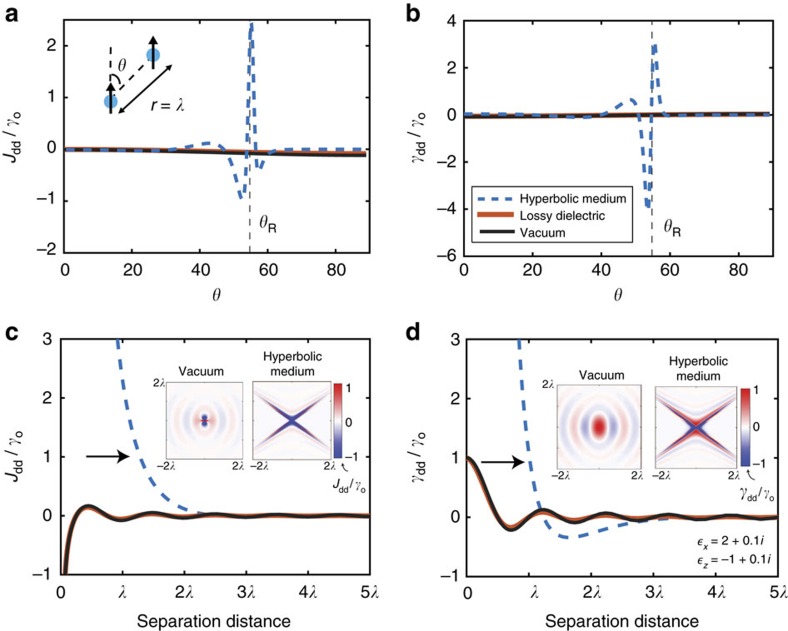
Manifestation of super-Coulombic interaction in hyperbolic media. Angular dependence of (**a**) CLS *J*_dd_ and (**b**) CDR *γ*_dd_ for two *z*-oriented dipoles in a lossy hyperbolic medium, lossy dielectric and vacuum. The CLS and CDR have large peaks near the resonance angle of the hyperbolic medium indicative of the super-Coulombic interaction, even for distances of a wavelength. Comparison of (**c**) CLS and (**d**) CDR at the resonance angle versus interatomic separation distance. The CLS and CDR both obey a 1/*r*^3^ power law dependence in the near-field due to the inclusion of absorption in the hyperbolic medium. It is noteworthy that the giant interactions start occuring at distances on the order of a wavelength (arrows) even in the presence of material absorption, which is in stark contrast to vacuum. The insets show the contrasting spatially-resolved (**c**) CLS and (**d**) CDR for vacuum and for a hyperbolic medium.

Figure 2 shows the result of the CLS and CDR for two *z*-oriented dipoles in a hyperbolic medium that includes material absorption. We compare the RDDIs with the conventional results of a lossy dielectric and vacuum. It is noteworthy that the RDDI peaks near the resonance angle *θ*_R_ as predicted theoretically. The spatial field plots in the insets clearly demonstrate the distinguishing features of the RDDI in a hyperbolic medium compared with vacuum. Figure 2c,d demonstrate the 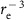 super-Coulombic spatial dependence along the resonance angle. It is noteworthy that the sign of the interaction is dependent on the orientation of the dipoles, as well as the relative position of the dipoles within the hyperbolic medium.

### Orientational dependence

**Figure 3:**
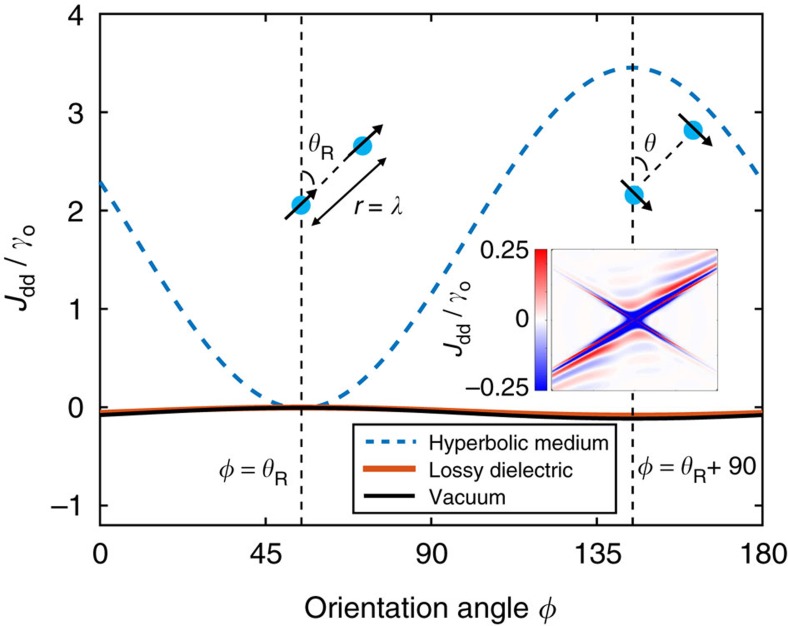
Unique orientational dependence of RDDI in hyperbolic media. The plot shows CLS versus orientation angle *φ* for two dipoles positioned along the resonance angle. The CLS is minimized when the dipoles are collinear with the the resonance angle and it is maximized when the dipoles are perpendicular to the resonance angle. The inset shows the asymmetric nature of the spatially-resolved *J*_dd_/*γ*_o_ when the dipoles are orthogonal to the resonance angle.

We now turn to the unique orientational dependence of the RDDI between two atoms positioned along the resonance angle *θ*_R_. In Fig. 3, we plot the normalized CLS of two atoms a full wavelength apart (*r*=*λ*) as a function of dipole orientation angle *φ*. The CLS has a minimum when *φ*=*θ*_R_ and a maximum when *φ*=*θ*_R_+*π*/2. Assuming that 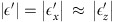, 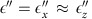, and 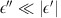, we find that the ratio between the maximum and minimum is


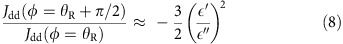


showing that it is proportional to the square of the figure of merit of the hyperbolic medium. In Fig. 3, we use the full Green's function to calculate the orientational dependence of the dipolar interaction in a hyperbolic medium with material absorption and find excellent agreement with the analytical expression.

### Resonance energy transfer

We now consider second-order super-Coulombic QED interactions between non-identical atoms arising from initial state preparation consisting of atom A in its excited state and atom B in its ground state, 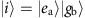. In the weak-coupling regime, an irreversible resonance energy transfer takes place, transferring a photon from atom A to atom B. This process is FRET and the transfer rate given by Fermi's golden rule is Γ_ET_=2*πħ*^−1^|*V*_dd_|^2^*δ*(*ħω*_a_−*ħω*_b_). Along the resonance angle, FRET is mediated by hyperbolic modes and the rate is given by


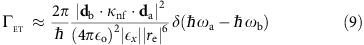


which shows a 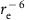 scaling dependence and giant enhancement—the key signature of second order super-Coulombic interactions in hyperbolic media.

### Casimir–Polder potential

In addition to the FRET rate, there is also a predicted frequency shift that comes from the initial state preparation 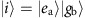. This is the excited-state Casimir–Polder potential, 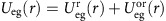, composed of a resonant and off-resonant contribution. The resonant excited-state Casimir–Polder potential is of the form 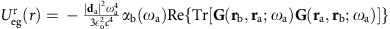 (ref. 34). We therefore predict that the excited-state energy potential will also diverge with a 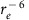 scaling dependence similar to the FRET rate.

**Figure 4:**
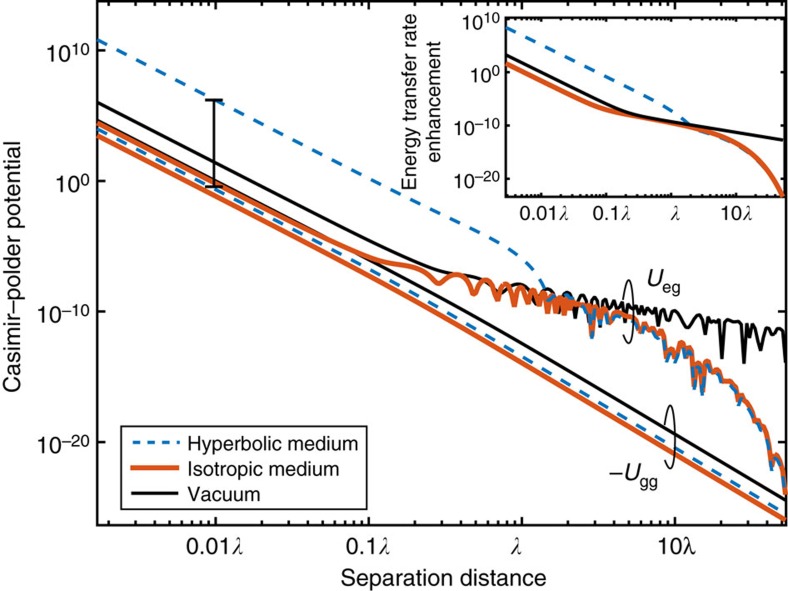
Ground-state and excited-state Casimir–Polder interaction energy in hyperbolic media. Casimir–Polder interaction energy between two ground-state atoms (*U*_gg_) and between an excited-state atom and ground-state atom (*U*_eg_) show fundamental differences when interacting in hyperbolic medium. *U*_eg_≫*U*_gg_, as resonant interactions lie completely within the bandwidth of hyperbolic dispersion and are strongly enhanced. The results are normalized to *U*_gg_ in vacuum, evaluated at the near-field interatomic distance of *r*_o_=*λ*/100. The inset shows the giant enhancement of the FRET rate, Γ_ET_, as compared with vacuum. The FRET rate is normalized to the vacuum energy transfer rate evaluated at *r*_o_.

Figure 4 shows the full numerical results for the second-order DDIs in a lossy hyperbolic medium, a lossy dielectric and vacuum. In the non-retarded regime 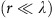, we clearly see the effect of the super-Coulombic interaction, which results in a large enhancement of the dipolar interactions *U*_eg_ and Γ_ET_ (shown in inset). The super-Coulombic enhancement occurs only along the asymptotes of the hyperboloid and is unrelated to the suppression of FRET rate of an ensemble of emitters near a conventional metallic surface or hyperbolic medium^35^,^36^,^37^.

It is interesting that the dispersive van der Waals interaction between two ground-state atoms does not diverge in a hyperbolic medium. Using fourth-order perturbation theory^38^, the interaction energy between two ground-state atoms is given by 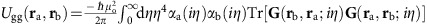, where *α*_A,B_(*ω*) is the isotropic electric polarizability of atom A or B. In the non-retarded limit, the dominant contribution is given by


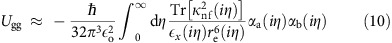


which reduces to the well-known free-space non-retarded van der Waals interaction energy when 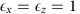. It is important to note that the integral is performed over the entire range of positive imaginary frequencies (*η*=*iω*). In general, the hyperbolic condition 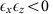 is only satisfied within a finite bandwidth of the electromagnetic spectrum. We therefore expect that it would not alter the broadband cumulative effect of the entire electromagnetic spectrum and, as a result, we predict that the ground state–ground state interaction energy will not diverge in a hyperbolic medium. From Fig. 4, it is also clear that the ground state–ground state Casimir–Polder potential *U*_gg_ does not show any type of enhancement for the hyperbolic medium, in agreement with our discussion. It is noteworthy that the distance scaling dependence in the non-retarded regions is in agreement with equations (9) and (10), as expected. In the retarded regime (*r*≫*λ*), the excited-state interactions *U*_eg_ and Γ_ET_ display an exponential damping behaviour due to material absorption, whereas the ground-state interaction *U*_gg_ displays the typical Casimir–Polder power law dependence, *r*^−7^ (Fig. 4).

## Discussion

In the following, we discuss multiple experimental platforms for hyperbolic media paving the way for the experimental demonstration of the long-range super-Coulombic interactions and unique many-body physics in hyperbolic media.

**Figure 5:**
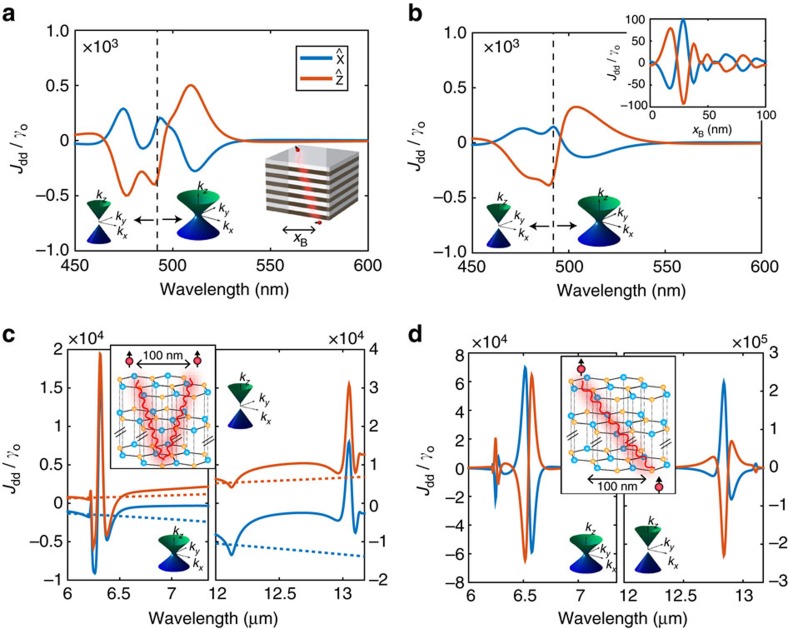
Giant long-range CLS in practical structures. (**a**,**b**) Plasmonic super-lattice in visible range and (**c**,**d**) natural hyperbolic medium in infrared range. (**a**) The effective hyperbolic model is compared with (**b**) a 40-layer multilayer system taking into account dissipation, dispersion and finite unit cell size. Atom A is 4 nm away from top interface, whereas atom B is adsorbed to the bottom interface with a horizontal displacement of *x*_b_=5 nm. The inset shows the CLS dependence on atom B's horizontal displacement when *λ*=550 nm. Good agreement is seen between the EMT model and practical multilayer design paving the way for an experimental demonstration of the super-Coulombic effect with cold atoms. CLS for (**c**) two atoms above h-BN and (**d**) two atoms across an h-BN structure; dashed lines denote bulk vacuum results. It is noteworthy that a smaller spontaneous emission rate 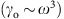 in the infrared range will contribute to a larger normalized CLS *J*_dd_/*γ*_o_. The orange and blue curves denote the two orientations of the transition dipole moment of the atoms. The total slab thickness for both structures is 100 nm.

Figure 5a–b propose a practical plasmonic super-lattice system to enhance atom-atom interactions taking into account the role of dissipation, dispersion and finite unit cell size. We show the large enhancement of CLS (*J*_dd_) for an effective medium model and compare it with a 40-layer structure consisting of Ag and TiO_2_ with a total slab thickness of 100 nm. For such a system, effective medium theory predicts a type I response 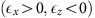 for wavelengths smaller than 492 nm and a type II response 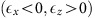 for wavelengths larger than 492 nm. Atom A is 4 nm away from the top interface (see Fig. 5 inset), whereas atom B is assumed to be adsorbed to the bottom interface. Atom B has a fixed horizontal displacement of *x*_b_=5 nm and therefore there is a fixed separation angle *θ*_o_ between atom A and atom B with respect to the normal to the interface. The two large peaks seen in Fig. 5 occur when the dispersive resonance angle *θ*_R_(*λ*) is equal to the fixed separation angle, that is, *θ*_R_(*λ*)=*θ*_o_ in agreement with theory. For the material system shown here, this occurs both in the type I and type II hyperbolic regions. The inset shows the directional sensitivity of the interaction as a function of atom B's horizontal displacement. It is noteworthy that accurate agreement between the effective medium model and the super-lattice structure is achieved when the unit-cell size is smaller than the separation distance between atom A and the top interface (Supplementary Note 1).

Figure 5c,d propose a two-dimensional van der Waals bonded natural material, hBN, as a candidate material to control optically active vibrational transitions between molecules, or electronic intersubband transitions between quantum wells. hBN is a natural hyperbolic medium in the mid-infrared spectral range. We show giant CLSs *J*_dd_ for the case of two atoms 10 nm away from the top interface of an h-BN structure, as well as for two atoms across an h-BN film. In the first case, the atom–atom interaction is due to a super-Coulombic ray-like interaction that reflects from the bottom interface (see insets). In the second case, the interatomic interaction is primarily due to a direct super-Coulombic interaction from atom A to atom B. Atom A is 10 nm above the top interface, whereas atom B is assumed to be adsorbed to the bottom interface. It is noteworthy that these long-range DDIs are seen equally in the type I hyperbolic region (*λ*∼12–13* *μm) and in the type II hyperbolic region (*λ*∼6–7 μm). We have used the experimentally verified permittivities for h-BN from Caldwell *et al*.^29^ for our numerical simulations.

**Figure 6:**
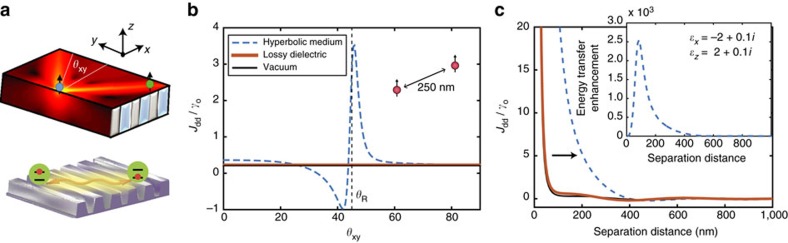
Super-Coulombic CLS above hyperbolic meta-surface. CLS, *J*_dd_, above hyperbolic metasurface with optic axis in the 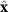 direction, calculated via dyadic Green function approach. Atom A and atom B are 2 nm above the interface. (**a**) Two-dimensional resonance cone on hyperbolic metasurfaces which causes giant in-plane long-range DDIs (**b**) CLS dependence on angle *θ*_*xy*_ of atom B for a fixed separation distance of *r*=*λ*/2=250 nm. The clear enhancement of the RDDI near the resonance angle *θ*_R_ is worth noting. (**c**) Separation distance dependence of CLS along the resonance angle *θ*_*xy*_=*θ*_R_. Inset shows giant FRET enhancement (>2,000) for separation distances of 100 nm in the metasurface plane.

Finally, Fig. 6 proposes a two-dimensional material system to enhance RDDIs, using hyperbolic metasurfaces. Our theoretical proposal provides additional future directions for designer metasurfaces based on graphene, black phosphorous, h-BN, gold/air or silver/air nanogratings^39^,^40^,^41^,^42^ (see Fig. 6a). We must emphasize that all of the experimental and theoretical studies thus far have focused on Purcell factor enhancements or the photonic spin-Hall effects. Here we propose hyperbolic metasurfaces to control many-body DDIs. Figure 6a shows the key difference from bulk hyperbolic media where a two dimensional resonance cone mediates giant long-range interactions due to in-plane hyperbolic dispersion (*x*–*y* plane anisotropy). In Fig. 6b, we show an enhancement of the CLS *J*_dd_ versus angle *θ*_*xy*_ of atom B. The angle *θ*_*xy*_ is defined with respect to the optic axis that lies parallel to the interface, such that 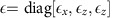. A clear enhancement is seen along the resonance angle *θ*_R_ compared with the vacuum and the dielectric half-space cases. Furthermore, when the position of atom B lies along the resonance angle 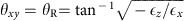 we find a clear order-of-magnitude enhancement in the CLS up to distances of 200 nm (Fig. 6b,c). Numerical simulations of the hyperbolic meta-surface were done using a dyadic Green function approach (Supplementary Note 2).

To summarize, we have revealed a class of singular excited-state atom–atom interactions in hyperbolic media that arise from a fundamental modification of the Coulombic near-field. The experimental observation of such effects will require careful isolation of medium-induced cooperative interactions between atoms from the effect of independent atoms interacting with the hyperbolic medium. Preliminary results have shown signatures of such interactions between molecules via FRET^43^. Future work should also focus on understanding the intricate role of non-locality^44^,^45^ on DDIs in hyperbolic media. Our work motivates the search for defect centers in natural hyperbolic media such as h-BN, where the interaction is mediated by hyperbolic phonon–polaritons. It should also motivate the study of unique many-body physics in atomic lattice quantum metamaterials with hyperbolic response^46^. Our work also paves the way for studies of long-range entanglement and self-organization^6^. It is also a first step towards cold-atom studies with hyperbolic meta-surfaces exhibiting unique effects that are not found in photonic crystals, waveguides or cavities.

## Methods

### Atomic system

In the following, we only consider the interaction between two identical atoms for the case of RDDIs. We then consider the interaction between two non-identical atoms for the case of second-order DDIs such as FRET and the excited-state Casimir–Polder interaction. For the simulations, we took the transition frequency of atom A to be *ω*_a_/2*π*=500 THz, whereas the transition frequency of atom B was *ω*_b_/2*π*=460 THz.

In the study, we provided equations for the interaction between two two-level systems for illustrative purposes. The generalized interaction between two *N*-level atoms can be easily extended with the general perturbation results. Furthermore, it is noteworthy that h-BN is considered due its high-quality factors and its low-loss phonon–polaritonic nature. The super-Coulombic effect will occur even in the presence of rapid dispersion in the dielectric constant of h-BN as long as the emitter linewidths are not significantly broader than the Reststrahlen bands of h-BN where optically active hyperbolic phonon-polaritons are found. For our simulations, representative values of loss and dielectric constants have been chosen from recent experiments in the mid-infrared spectral range.

### Perturbation theory

All DDIs can be calculated from the transition matrix element:


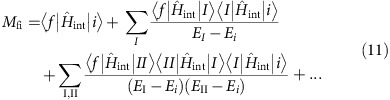


where the summation in the second- and higher-order terms runs over all possible intermediate states; the summation can be replaced by integration for the case of continuum states. The energy level shift of the initial state 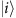 is then given by


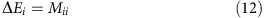


where it is understood that the principal value is taken during the integration of continuum intermediate states. The probability transition rate from initial state 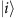 to final state 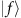 is given by Fermi's Golden rule


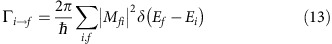


where the summation runs over all initial and final states.

#### Resonant dipole–dipole interaction

We now consider the interaction between two identical atoms, labelled atom A and atom B, respectively. The probability transition rate between state 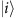 and 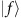 is found through equation (13) to give the CDR


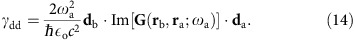


Assuming the dipole moments of both atoms are oriented along the same direction, the total decay rate of two identical atoms will be *γ*_tot_=*γ*_a_±*γ*_dd_, where *γ*_a_ is the bare spontaneous emission rate of atom A (or atom B). The initial state is 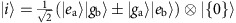 and final state 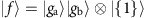. It is noteworthy that 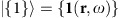 represents the single-photon Fock state with position **r** and frequency *ω*.

The first-order dipole–dipole frequency shift of initial state 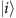 is then found through evaluation of equation (12), which results in a resonant and off-resonant contribution 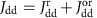, specified by


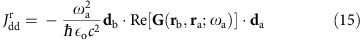


and


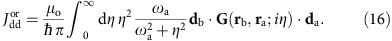


In the Letter, we only retain the resonant contributions (4) and (5), as they give rise to the super-Coulombic DDIs. The results agree with those of ref. 47.

#### Resonance energy transfer rate

Using equation (3), the resonance energy transfer rate between state 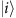 and 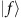 can be calculated to give


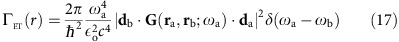


where we have taken 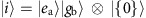 and final state 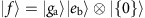 (ref. 48).

### Excited state–ground state interaction

The excited-state Casimir–Polder potential is given by^34^


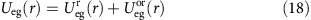


where the resonant component is


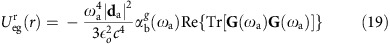


and off-resonant component is given by


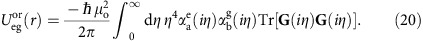



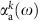
 is the isotropic electric polarizability of atom A in the *k*th energy eigenstate, defined as


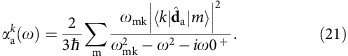


#### Ground state–ground state interaction

The ground state–ground state Casimir–Polder potential is given by^38^


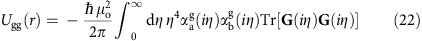


which is applicable in the retarded and non-retarded regimes. It is noteworthy that we have dropped the spatial coordinate dependence of the Green function in equations (19), (20) and (22).

It is worth noting that for the numerical calculation of the integrals, we considered an anisotropic medium with a Lorentz resonance parallel to the optic axis with parameters *ω*_*Pz*_/2*π*=550 × 10^12^ Hz, *ω*_*Tz*_/2*π*=450 × 10^12^ Hz and *γ*_*z*_=0.01*ω*_*Pz*_, and a Lorentz resonance perpendicular to the optic axis with parameters *ω*_*Px*_/2*π*=770 × 10^12^ Hz, *ω*_*Tx*_/2*π*=600 × 10^12^ Hz and *γ*_*z*_=0.01*ω*_*Px*_. The isotropic medium had the same relative permittivity as the *x* axis of the anisotropic medium.

### Applicability of perturbation theory

It is noteworthy that the perturbative formalism used in this work is strictly applicable for the case of finite absorption with a sufficiently large interatomic separation distance. This is in agreement with our simulations for practical experimental systems such as plasmonic super-lattices and hyperbolic metasurfaces. For the case of low losses and extremely short separation distances, a non-perturbative treatment will be required to treat the dipole–dipole singularity in a self-consistent manner. It is also noteworthy that the presence of emitters do not alter the hyperbolic polaritonic branches in the weak coupling limit.

### Green function in a uniaxial medium

The Green tensor is the unique solution to the homogeneous Helmholtz equation with permittivity tensor 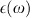,


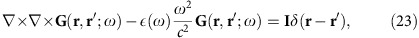


and radiation condition **G**(**r**, **r**′; *ω*)=0 for |**r**−**r**′|→∞. The coordinate-free form of the Green function is given by^49^


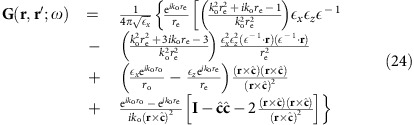


where we have fixed the spatial coordinate of the source at the origin, that is, **r**′=0. It is noteworthy that this Green function is only applicable when **r**≠**r**′, as we have excluded the singularity term that occurs when **r**=**r**′.

### Data availability

The data that support the findings of this study are available from the corresponding author upon request.

## Additional information

**How to cite this article:** Cortes, C. L. *et al*. Super-Coulombic atom-atom interactions in hyperbolic media. *Nat. Commun.* **8,** 14144 doi: 10.1038/ncomms14144 (2017).

**Publisher's note**: Springer Nature remains neutral with regard to jurisdictional claims in published maps and institutional affiliations.

## Supplementary Material

Supplementary Information Supplementary Figure 1, Supplementary Table 1, Supplementary Notes 1-2 and Supplementary References.

## Figures and Tables

**Figure 1:**
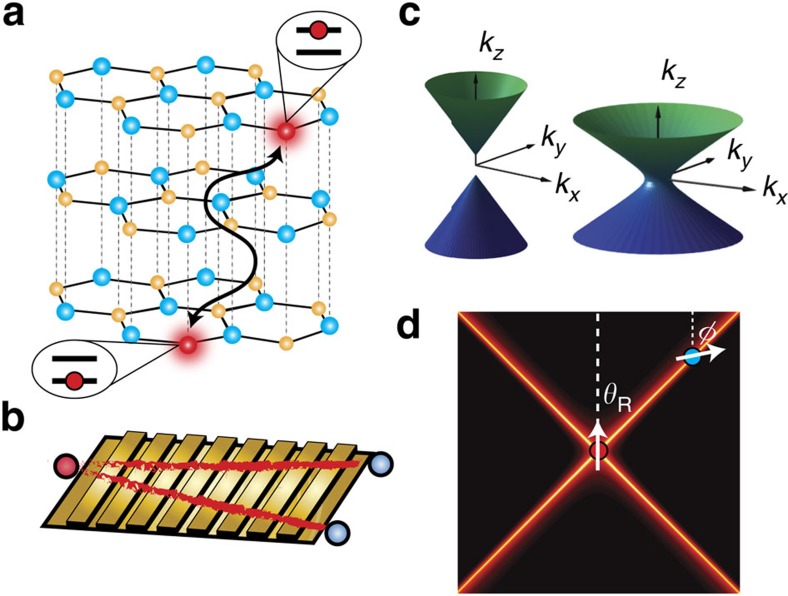
Overview of super-Coulombic interaction. The proposed long-range super-Coulombic DDI may be observed (**a**) between single-photon defect centres in natural hyperbolic media (for example, h-BN, Bi_2_Se_3_ and Bi_2_Te_3_) or (**b**) between ultra-cold atoms trapped above a hyperbolic meta-surface. (**c**,**d**) The super-Coulombic interaction occurs over a broad range of frequencies along the resonance angle of a hyperbolic medium and causes the effective interaction distance to approach zero irrespective of the physical distance.
