# Supplementary material for: Super-Coulombic atom–atom interactions in hyperbolic media
Source: Nat Commun. 2017 Jan 25;8:14144. doi: 10.1038/ncomms14144 (PMC5288497; doi:10.1038/ncomms14144)
Supplement: Supplementary Information — Supplementary Figure 1, Supplementary Table 1, Supplementary Notes 1-2 and Supplementary References. [file ncomms14144-s1.pdf]

Supplementary Information

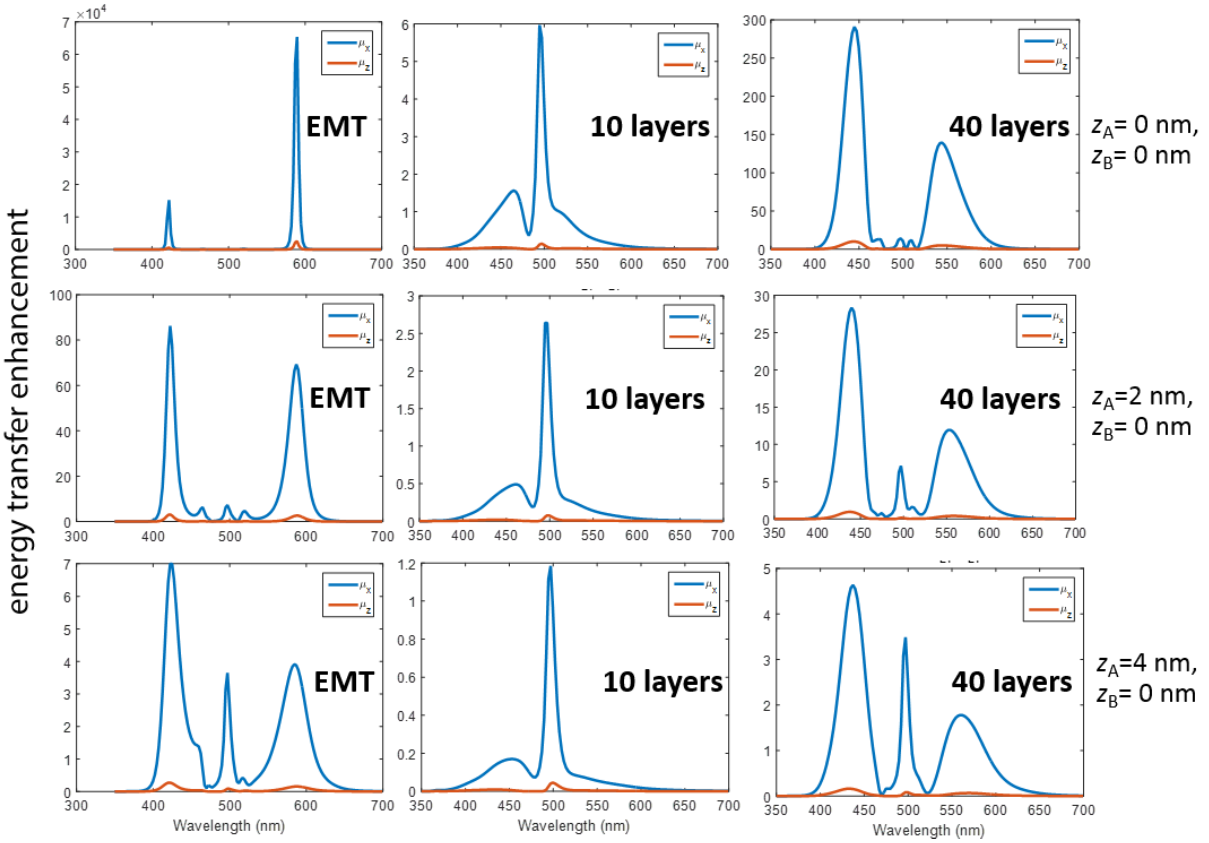

Supplementary Figure 1. Comparison of plasmonic super-lattice and effective medium theory as a function of sublayer thickness, total number of layers, and donor separation distance from top interface.

**Platform**	Circuit QED	Ultra-cold atoms		Self-assembled quantum dots	**Our materials approach**
		photonic crystal	optical trap		
**dipole-dipole interaction method**	transmission line waveguide	band-edge waveguide (1-D)	conventional near-field dipole interaction	conventional far-field dipole interaction	controlled by *k*-surface engineering of hyperbolic polaritons
**Temperature requirement**	low temp. $\ll 1$ K	low temp. $\ll 1$ mK	low temp. $\ll 1$ mK	low temp. ~ 2 K	room temp. due to broadband effect
**Scaling** J_{dd}	(radiative) $\sim \sin(k_o r)$	(radiative) $\sim \sin(k_o r)$	(non-radiative) $\sim 1/r^3$	(radiative) $\sim \cos(k_o r)/r$	(Super-Coulombic) $\sim 1/(N(\theta)r)^3$, $N(\theta) \to 0$
**Typical spatial range**	few wavelengths $\sim \lambda$	few wavelengths $\sim \lambda$	extreme near-field $\sim \lambda/100$	sub wavelength $\sim \lambda/2$	>10x beyond near-field range; 10^2 - 10^3 magnitude increase from other materials

Supplementary Table 1: Comparison of Super-Coulombic interaction with established techniques for controlling dipole-dipole interactions.

Supplementary Note 1. Plasmonic Super-Lattice analysis

In this note, we provide numerical simulations comparing a practical plasmonic super-lattice structure with the corresponding effective medium theory. We consider the effect of sublayer thickness and total number of layers in the super-lattice metamaterial structure.

In supplementary Fig. 1, we plot the energy transfer enhancement for two dipoles on either side of a slab with a fixed slab thickness of 100 nm. By controlling the total number of layers of the super-lattice, we show the net effect of sublayer thickness and unit-cell size. We also vary the separation distance z_A of atom A from the top interface. Atom B is assumed to be adsorbed to the bottom interface. Note that the super-lattice structure has a better match with EMT as the unit-cell size decreases and the total number of layers increases. This is a result of the wave-vector cut-off that is imposed by the finite unit-cell size.

Supplementary Note 2. Dyadic Green function for hyperbolic meta-surface

Here, we provide details for the calculation of resonant dipole-dipole interactions above a hyperbolic meta-surface. The hyperbolic meta-surface is modeled as uniaxial medium half-space with an optic axis c parallel to the interface, $\hat{\boldsymbol{c}} = \hat{\boldsymbol{x}}$. The dyadic Green function can be decomposed in terms of a bulk and scattered term in the upper half-space (z > 0) and a scattered-only term in the lower half-space (z < 0)

$$\mathbf{G}(\mathbf{r},\mathbf{r}',\omega) = \mathbf{G}_o(\mathbf{r},\mathbf{r}',\omega) + \mathbf{G}^r(\mathbf{r},\mathbf{r}',\omega) \qquad \text{for} \quad z, z' > 0 \qquad (1)$$

$$\mathbf{G}(\mathbf{r},\mathbf{r}',\omega) = \mathbf{G}^t(\mathbf{r},\mathbf{r}',\omega) \qquad \text{for} \quad z < 0, z' > 0 \qquad (2)$$

The source medium is assumed to be vacuum ($\varepsilon_1 = 1$), while the uniaxial half-space has permittivity $\boldsymbol{\varepsilon} = \mathrm{diag}[\varepsilon_x, \varepsilon_z, \varepsilon_z]$. Applying a plane-wave expansion, the reflected and transmitted dyadic Green functions for two z-oriented dipoles take the form

$$G^r_{zz}(\mathbf{r}) = \frac{i}{8\pi^2 k_1^2}\int dk_\rho d\phi \, \frac{k_\rho^3}{k_{z1}} r_{pp}(k_\rho,\phi)\, e^{ik_\rho\rho\cos(\phi-\theta)+ik_{z1}(z+d)} \qquad (3)$$

$$G^t_{zz}(\mathbf{r}) = \frac{i}{8\pi^2 k_1 k_2}\int dk_\rho d\phi \, \frac{k_\rho^3}{k_{z1}} \left[t_{op}(k_\rho,\phi) k_2 \sin\phi e^{-ik_{zo}z} + t_{ep}(k_\rho,\phi) k_{ze}\cos\phi e^{-ik_{ze}z}\right] e^{ik_\rho\rho\cos(\phi-\theta)+ik_{z1}d} \qquad (4)$$

where $k_1 = \sqrt{\varepsilon_1}\omega/c$, $k_2 = \sqrt{\varepsilon_z}\omega/c$ and d is the distance away from the interface of the donor dipole. Note that we have used the cylindrical coordinates

$$k_x = k_\rho\cos\phi \quad , \quad k_y = k_\rho \sin\phi$$
$$x = \rho\cos\theta \quad , \quad y = \rho\sin\theta.$$

and also defined $k_{z1} = \sqrt{k_1^2 - k_\rho^2}$ for the upper half-space, and $k_{zo} = \sqrt{k_2^2 - k_\rho^2}$, $k_{ze} = \sqrt{\varepsilon_x\omega^2/c^2 - k_y^2 - k_x^2\varepsilon_x/\varepsilon_z}$ as the ordinary and extraordinary wave contributions in the lower half-space respectively. The p-polarization to p-polarization reflection coefficient takes the form

$$r_{pp} = -\frac{\epsilon_z k_y^2 k_o^3 (k_{z1} + k_{ze})(\epsilon_1 k_{zo} - \epsilon_z k_{z1}) + k_x^2 k_{zo} k_o (k_{z1} + k_{zo})(\epsilon_1 k_{zo}^2 - \epsilon_z k_{z1} k_{ze})}{\sqrt{\epsilon_z}\Delta(k_x^2 + k_y^2)\sqrt{(k_y^2 + k_{zo}^2)(k_y^2 + k_{ze}^2)}} \quad (5)$$

while the p-polarization to ordinary- and extraordinary- polarization transmission coefficients take the form

$$t_{op} = \frac{2k_{z1}k_y k_o^2}{\Delta}\frac{(k_{z1} + k_{ze})\sqrt{\epsilon_1 \epsilon_z}}{\sqrt{(k_x^2 + k_y^2)(k_{ze}^2 + k_y^2)}} \quad (6)$$

$$t_{ep} = \frac{2k_{z1}k_{zo}k_1 k_x}{\Delta}\frac{k_{z1} + k_{zo}}{\sqrt{(k_x^2 + k_y^2)(k_y^2 + k_{zo}^2)}} \quad (7)$$

where

$$\Delta = \frac{k_x^2 k_{zo} k_o (k_{z1} + k_{zo})(\epsilon_1 k_{zo}^2 + \epsilon_z k_{z1} k_{ze}) + \epsilon_z k_y^2 k_o^3 (k_{z1} + k_{ze})(\epsilon_1 k_{zo} + \epsilon_z k_{z1})}{\sqrt{\epsilon_z}(k_x^2 + k_y^2)\sqrt{k_{zo}^2 + k_y^2}\sqrt{k_{ze}^2 + k_y^2}} \quad (8)$$

Supplementary References

[1] Luigi Frunzio, Andreas Wallraff, David Schuster, Johannes Majer, and Robert Schoelkopf. *Fabrication and characterization of superconducting circuit qed devices for quantum computation. IEEE transactions on applied superconductivity*, 15(2):860-863, (2005).

[2] A Goban, C-L Hung, JD Hood, S-P Yu, JA Muniz, O Painter, and HJ Kimble. *Superradiance for atoms trapped along a photonic crystal waveguide*. Physical review letters, 115(6):063601, (2015).

[3] Sylvain Ravets, Henning Labuhn, Daniel Barredo, Lucas B_eguin, Thierry Lahaye, and Antoine Browaeys. *Coherent dipole-dipole coupling between two single rydberg atoms at an electrically-tuned forster resonance*. Nature Physics, 10(12):914-917, (2014).

[4] Michael Scheibner, Thomas Schmidt, Lukas Worschech, Alfred Forchel, Gerd Bacher, Thorsten Passow, and Detlef Hommel. *Superradiance of quantum dots*. Nature Physics, 3(2):106-110, (2007).
